# FMRI to probe sex-related differences in brain function with multitasking

**DOI:** 10.1371/journal.pone.0181554

**Published:** 2017-07-31

**Authors:** Melanie Tschernegg, Christa Neuper, Reinhold Schmidt, Guilherme Wood, Martin Kronbichler, Franz Fazekas, Christian Enzinger, Marisa Koini

**Affiliations:** 1 Institute of Psychology, Karl-Franzens-University Graz, Graz, Austria; 2 Centre for Cognitive Neuroscience and Department of Psychology, University of Salzburg, Salzburg, Austria; 3 Department of Neurogeriatrics, Medical University of Graz, Graz, Austria; 4 Neuroscience Institute and Centre for Neurocognitive Research, Christian-Doppler-Klinik, Paracelsus Medical University Salzburg, Salzburg, Austria; 5 Department of Neurology, Medical University of Graz, Graz, Austria; 6 Divisions of Neuroradiology, Department of Neurology, Medical University of Graz, Graz, Austria; 7 BioTechMed-Graz, Graz, Austria; Universitatsklinikum Tubingen, GERMANY

## Abstract

**Background:**

Although established as a general notion in society, there is no solid scientific foundation for the existence of sex-differences in multitasking. Reaction time and accuracy in dual task conditions have an inverse relationship relative to single task, independently from sex. While a more disseminated network, parallel to decreasing accuracy and reaction time has been demonstrated in dual task fMRI studies, little is known so far whether there exist respective sex-related differences in activation.

**Methods:**

We subjected 20 women (mean age = 25.45; SD = 5.23) and 20 men (mean age = 27.55; SD = 4.00) to a combined verbal and spatial fMRI paradigm at 3.0T to assess sex-related skills, based on the assumption that generally women better perform in verbal tasks while men do better in spatial tasks. We also obtained behavioral tests for verbal and spatial intelligence, attention, executive functions, and working memory.

**Results:**

No differences between women and men were observed in behavioral measures of dual-tasking or cognitive performance. Generally, brain activation increased with higher task load, mainly in the bilateral inferior and prefrontal gyri, the anterior cingulum, thalamus, putamen and occipital areas. Comparing sexes, women showed increased activation in the inferior frontal gyrus in the verbal dual-task while men demonstrated increased activation in the precuneus and adjacent visual areas in the spatial task.

**Conclusion:**

Against the background of equal cognitive and behavioral dual-task performance in both sexes, we provide first evidence for sex-related activation differences in functional networks for verbal and spatial dual-tasking.

## Introduction

Differences between men and women concerning cognitive processes and related brain-networks arouse public and scientific interest. Interestingly, in the western world it seems to represent common sense that women perform better in completing multiple simultaneously presented tasks than men, i.e. are more capable of ‘multitasking’. However, this view lacks support from current scientific research [1].

During dual-tasks, people perform two different tasks alternately [2]. The ‘primary task’ is presented and followed by a secondary task after a certain stimulus onset asynchrony (SOA) [3]. According to the ‘response selection bottleneck theory’ it is assumed that the mental processing (decision making) of the ‘primary task’ has to be finished before processing of the ‘secondary task’ can be processed. Pashler (1994) postulates that, at a given point of time, only one decision can be made. Accordingly, the average reaction time (RT) increases in the inverse proportion of SOA duration [3].

Several studies used brain-imaging methods such as fMRI and PET to investigate brain activation during performance of a dual-task paradigm [4–9]. The findings suggested a more disseminated network parallel to increasing task requirements [4,8], primarily in the inferior frontal lobe [5,8–10], the posterior and inferior parietal lobes [11], the cingulate gyrus [12] and the lateral prefrontal cortex [9,10,12].

More specifically, sex-related differences in such cognitive performance and neuronal processing have received increasing interest in the last years [13–15]. In this context, mental rotation tasks in 3-dimensional presentation and word fluency tasks show clear behavioral performance differences between both sexes [16]. In contrast, cognitive sex differences in verbal tasks do not appear to be as strong (compared to spatial abilities), although word fluency seems to be the construct yielding the most robust differences [17]. Working memory offers another example of cognitive sex differences, with women performing better in terms of reaction times and errors than men [18]. Since working memory is one of the necessary functions to enable dual- and multitasking [7,19,20] such sex differences in working memory could explain possible differences in dual tasking between men and women.

Paridon and Kaufmann [1] were the first to investigate sex-related differences in multitasking and failed to report behavioral sex differences. In contrast, another recent study combining an executive function task with a task measuring spatial ability identified sex differences in multitasking [21]. These findings provide first evidence that spatial ability might be one of the major abilities that modulate sex differences in multitasking, but investigations focusing on underlying neurophysiological differences between men and women in dual tasking are scarce.

The few neuroimaging studies on sex-related differences in verbal, spatial and working memory tasks yielded largely consistent results [22–25]. Sex-related lateralization effects or network differences were observed in different cognitive tasks [22]. Regarding working memory, women demonstrate a more left lateralized activation of the lateral prefrontal cortex, the parietal cortices, and the caudate, while men show a more right lateralized activation [26].

However, it is not clear yet whether men and women process verbal and spatial dual-tasks differently. The aim of the present study thus was to identify such potential sex-related differences in cerebral activation patterns during dual-tasking. For this purpose, we generated a combined verbal and spatial fMRI paradigm not targeting on identifying differences on single task level.

## Materials and methods

### Subjects

Forty age-matched (mean = 26.5 years; SD = 4.73; range = 18–36), right-handed, German native speaking healthy participants (20 female, 20 male) took part in this experiment. The ethics committee on human experimentation of the Medical University of Graz had approved the study. Subjects gave written informed consent.

### Neuropsychological assessment

The following neuropsychological tests were used to control for cognitive performance: three subtests of the verbal “Intelligenz Struktur Test” (IST-2000R, German versions of an intelligence test involving completing sentences, analogy and similarities) and spatial IST-2000R (cubes, figure selection and matrices) [27], the Stroop Task (color word interference test) [28] and the Trail Making Test (version A and B) [29].

### FMRI dual task paradigm

**Fig 1:**
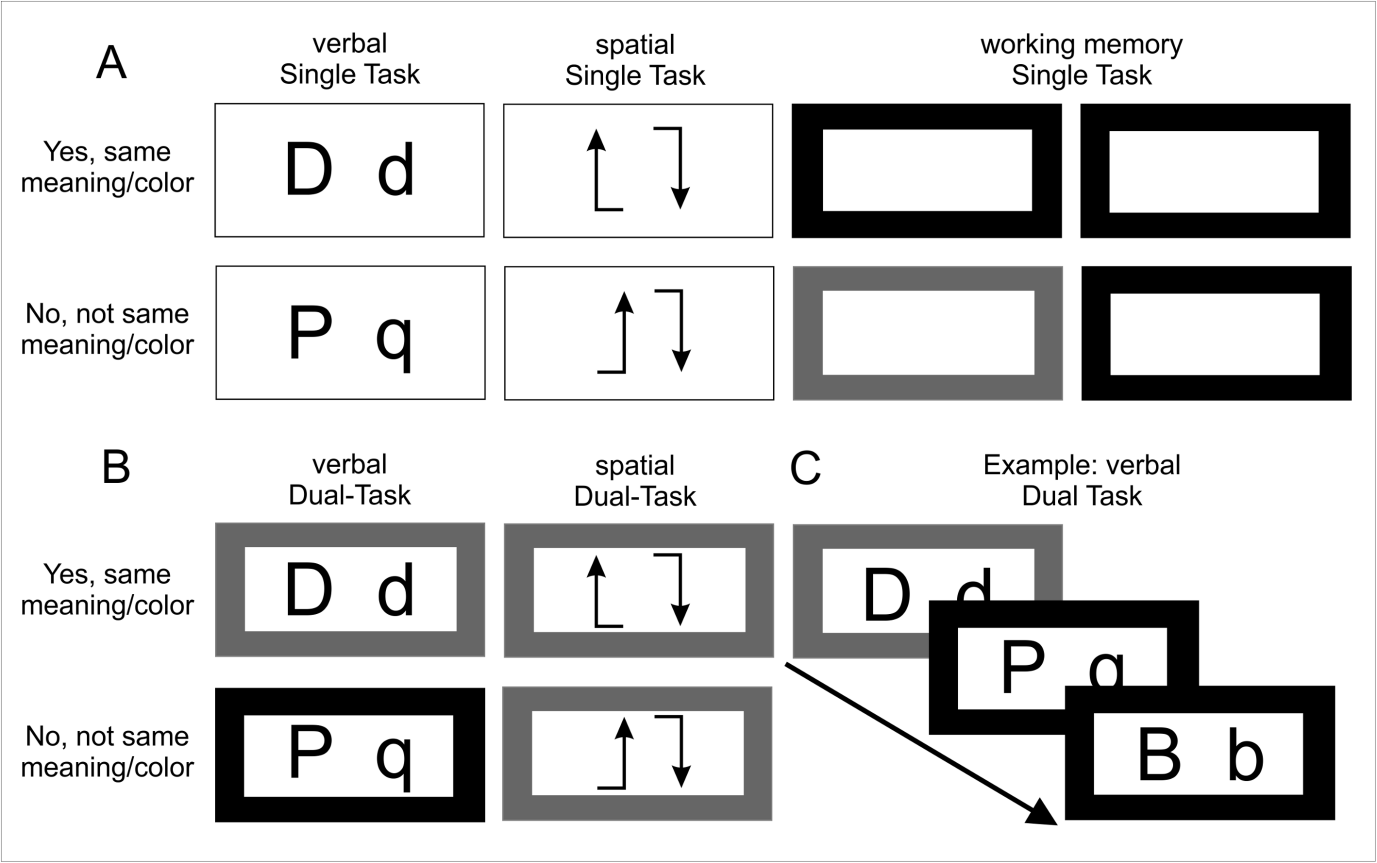
Dual-task paradigm: A: verbal-, spatial-, and working memory single-task. Yes/No decisions over equality for the verbal task in terms of meaning, for the spatial task in term of rotation and for the working memory in terms of its color in a one-back task decision. B: Combination of working-memory task, and verbal or spatial single-task. C: Example of the verbal dual-task. Deciding over both tasks with a stimulus-onset-asynchrony (SOA) of 200 ms.

The paradigm consisted of 30-second active blocks (single task and dual task), alternately presented with 24-second rest phases characterized by a crosshair in the middle of the display. A verbal single task and a spatial single task were each combined with a one-back task, yielding a total of four conditions: (1) a verbal single task, (2) a spatial single task, (3) a verbal dual task, and (4) a spatial dual task. In addition, a working memory task was implemented as a single task (Fig 1). Participants completed five blocks of each dual task-condition and four blocks of each single-task condition in pseudo-randomized order (duration: 1230 seconds).

Participants had to decide on equality or disparity of letters during the verbal single task (e.g. D and d: different appearance but same meaning; P and q: different meaning) [24]. The following letters were used: D, d, P, p, Q, q, B, b.

During the spatial task, participants had to compare two arrows and decide whether they were matching or not after mental rotation (Fig 1, A = single task; B = dual-task; C = example) [24].

During the working memory one-back-task (secondary task), participants had to decide whether the current presented frame on the display had the same color as the frame presented one image before.

For all three single-tasks, participants had to press with both index fingers simultaneously using an fMRI compatible response box for a ‘Yes, same meaning/color’ and both middle fingers for a ‘No, not the same meaning/color’ response. This approach was chosen to take into account increased motor-related activation changes during dual task blocks. In the dual-task condition, people had to perform the primary task with the right response box and the secondary task with the left response box (index or middle finger, respectively). Stimuli were presented for 3000 ms and in the dual task condition the secondary task was presented with a fixed SOA of 200ms.

### Data acquisition

Images were acquired using a 3T MRI scanner (Tim Trio, Siemens, Erlangen, Germany) with a 32-channel coil. The automatic motion control was switched off. The following gradient echo-planar imaging sequences were obtained: 418 T2*-weighted volumes, TR = 3000, TE = 30ms, FA = 90°, matrix size = 64x64, voxel size = 3.0x3.0x3.0mm. The first two volumes were excluded to allow for T1 equilibration. The high-resolution T1 scan had the following parameters: 3D-MPRAGE; TR = 1900ms, TE = 2.6ms, TI = 900ms; 1.0x1.0x1.0mm.

### Data analysis

Functional data were analyzed using FEAT (Version 5.63, www.fmrib.ox.ac.uk/fsl). The following preprocessing steps were applied: motion correction (MCFLIRT), non-brain removal (BET), spatial smoothing (FWHM with a kernel of 5mm), and normalization (MNI-space), FSL-template of 152 subjects.

High pass temporal filtering was selected for 63s. FILM provided time-series with statistical analysis with local autocorrelation correction. FLIRT was used for registration to high-resolution/standard images. Higher-level-analyses were done using FLAME 1. A Z statistic threshold of *z* = 2.3 (verbal and spatial single-tasks; verbal and spatial dual-tasks) was used. Correction for multiple comparisons was conducted by using FDR in FSL.

During first level analyses, all active conditions (single-task and dual-task conditions) were compared to baseline. Motion parameters were used as a covariate of no interest. Higher level analyses were conducted for Dual-Task < Single Task. Furthermore we conducted the contrast Men > Women, as well as Women > Men for both “Dual-Task < Single-Task”- contrasts (verbal and spatial). Two participants had to be excluded from the analyses due to severe movement artifacts (pre-defined as motion > 3mm). For higher-level statistics, the sample thus comprised 19 men and 19 women.

### Contrast masking

Activation patterns of all study participants (i.e. of both men and women) for the verbal and the spatial dual-task condition (dual-task > single-task) were used to mask the activation patterns for the group comparison between men and women in order to ensure that only regions involved in the given task can yield significant sex differences. A Z statistic threshold for the masking of z = 2.3 was used.

### Statistical analyses

The Statistical Package of Social Sciences (SPSS, Version 21) was used for inter-group comparison of behavioral data. Normal distribution was assessed with the Shapiro-Wilk Test. Whenever appropriate t-test was used for group comparisons. If data were not normally distributed Mann-Whitney U and Wilcoxon Test was used.

## Results

### Neuropsychological results and demographics

Verbal and spatial IQ measured by six subtests of the IST2000R were comparable for men and women (verbal IQ: t(38) = 0.173, *p* = 0.863; spatial IQ: t(38) = -0.746, *p* = 0.461; compare online supplemental S1 Table). Also the executive functions measured by the Stroop Task, subtest interference and the Trail Making Test B did not show differences between men and women (U = 137.0, p = 0.091) U = 146.5, p = 0.149). Moreover, attention, as measured by two subtests of the Stroop task (Reading words: U = 187.0; p = 0.738, Naming colors: U = 163.5, p = 0.327) and the Trail Making Test A (U = 160.0, p = 0.289) did not reveal differences between sexes. Men and women did not differ regarding age (U = 136.0, p = 0.086) or educational level measured in years of education (U = 170.0, p = 0.429, Table 1).

**Table 1:** Demographics and descriptive statistics for all experimental conditions.

	Women	Men	p-value
Demographics			
Age in Years	25.45±5.24	27.55±4.01	0.086
Education in Years	17.03±2.93	17.75±3.31	0.429
Neuropsychological Tests			
Stroop Reading words	26.66±2.87	26.73±4.41	0.738
Stroop Naming colors	39.87±4.56	44.53±13.63	0.327
Stroop interference	60.45±8.29	67.90±14.74	0.091
Verbal IST-2000R	109.05±11.77	108.40±11.94	0.863
Spatial IST-2000R	102.15±9.24	104.35±9.42	0.461
Trail Making A	21.70±7.23	20.90±8.36	0.289
Trail Making B	48.62±16.92	55.31±20.74	0.149
Reaction Time (ms)			
Verbal Single-Task	983.55±111.80	983.40±212.39	0.947
Verbal Dual-Task	1472.05±192.17	1380.75±394.88	0.529
Spatial Single-Task	1317.20±177.92	1241.30±197.75	0.210
Spatial Dual-Task	1122.45±274.89	1203.15±173.77	0.274
Working Memory Single-Task	791.90±121.08	721.45±150.52	0.111
Working Memory Verbal Dual-Task	1424.55±323.57	1432.20±231.51	0.758
Working Memory Spatial Dual-Task	1364.40±375.12	1503.40±266.65	0.529
Accuracy Rates			
Verbal Single-Task	9.80al S5	9.69al S5	0.211
Verbal Dual-Task	7.37al D5	8.07al 54	0.478
Spatial Single-Task	8.66±6.74	8.61±1.15	0.620
Spatial Dual-Task	6.44ial89	7.18i2.06	0.478
Working Memory Single-Task	8.39ing90	8.69ing 0	0.253
Working Memory Verbal Dual-Task	5.83ing33	6.76ing 2	0.253
Working Memory Spatial Dual-Task	5.73±2.95	6.49±2.01	0.495

### Behavioral performance in the scanner

Overall, RT and decreasing accuracy in the dual-task conditions compared to the single-task conditions for primary and secondary tasks. There is no sex-related difference in the reaction time or the accuracy in dual-task or single-task (Table 1). The reaction time for the working memory task increased significantly in the verbal and spatial condition compared to the single-task condition (verbal: Z = -5.256, p<0.001, spatial: Z = -5.202, p<0.001). Accuracy for the working memory task was lower in the dual-task conditions compared to the single-task condition (verbal: Z = -5.512, p<0.001; spatial: Z = -5.196, p<0.001). Also, the primary tasks showed increased reaction time and less accuracy in the dual-task condition in the whole sample. The verbal dual-task reaction time increased compared to the single-task (Z = -5.377, p<0.001), and the accuracy decreased (Z = -5.499, *p* < 0.001). The spatial dual-task reaction time (Z = -2.61, p = 0.009) was significantly higher and the accuracy was lower (Z = -4.489, *p* < 0.001) than in the spatial single-task condition.

**Fig 2:**
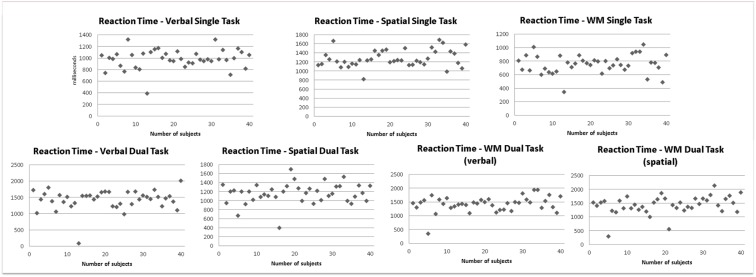
Reaction times for all single-tasks and dual-tasks. The scatter plots show the actual data of all 38 participants for visual verification of the distribution.

Considering sex, no differences in behavioral performance were found. Men and women showed no significant differences in reaction times and accuracy in all single-tasks (working memory task, verbal single-task, spatial single-task) and dual-task conditions (verbal dual-task, spatial dual-task). Also the secondary task demonstrated no differences between the sexes during the dual-task condition (see Fig 2 for the actual distribution of the behavioral performance).

### Functional MRI data: Task specific activation

Whole brain analyses independent of sex for the verbal dual-task vs. verbal single-task revealed a network comprising the right superior frontal gyrus, the right medial frontal gyrus, the right precuneus, the lateral occipital cortex, the right occipital fusiform gyrus, the left superior frontal gyrus and the left middle frontal gyrus.

**Fig 3:**
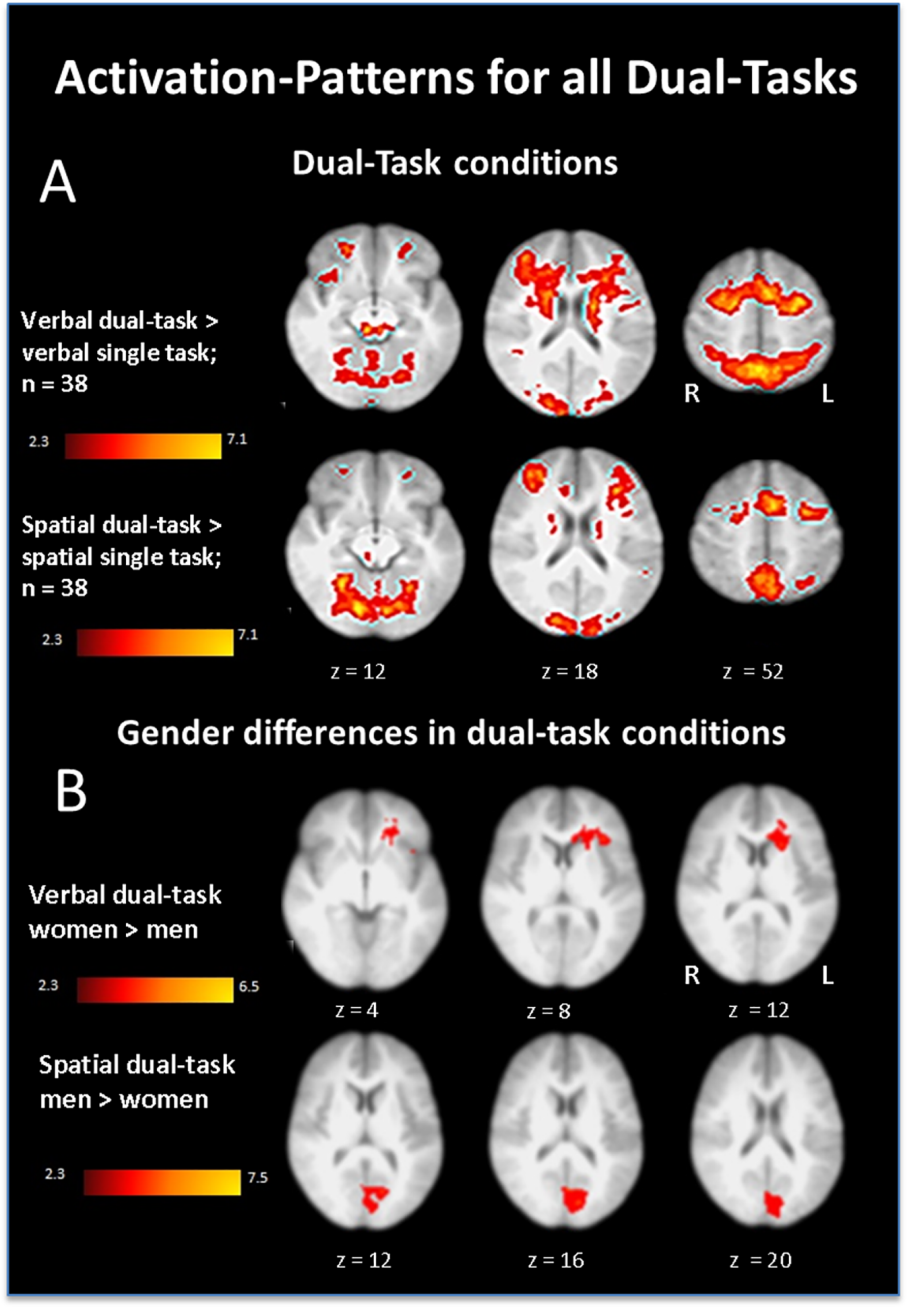
Activation-patterns for all verbal and spatial dual-tasks compared with single-tasks. (A) Activation patterns for verbal and spatial dual-task compared with single-task over all subjects. (B) Sex related differences for verbal and spatial dual-task compared with single-task. Orientation: right /left.

The comparison of the spatial dual-task vs. spatial single-task indicated increased activation for the dual-task condition in the following regions: the left inferior frontal gyrus, the bilateral middle frontal gyrus, the left orbitofrontal gyrus, the right anterior cingulate and left anterior paracingulate as well as the bilateral precuneus and left lateral occipital cortex. Subcortical regions such as the left thalamus, the left caudate, the left insula and left putamen were also identified as active for the spatial dual-task condition compared with the spatial single-task condition. See Fig 3A, Tables 2 and 3 for the whole brain analyses of both dual-task conditions in comparison to the single-task conditions.

**Table 2:** Significant activation patterns (local maxima) for the verbal dual-task condition.

Brain Regions	Verbal Dual-Task > Verbal Single-Task			
	x	y	z	Z-score
Juxtapositional Lobule Cortex	-8	4	60	8.29
Superior Frontal Gyrus	24	-2	56	7.69
Middle Frontal Gyrus	32	-2	56	7.65
Superior Frontal Gyrus	-16	-6	58	7.52
Precentral Gyrus	-28	-6	54	7.5
Paracingulate Gyrus	-4	10	50	7.5
Lateral Occipital Cortex	-20	-60	60	8.13
Precuneus Cortex	0	-64	54	7.9
	8	-68	48	7.71
	8	-60	52	7.58
	10	-60	46	7.29
	18	-62	42	7.21
Lateral Occipital Cortex	30	-64	-24	7.35
Occipital Fusiform Gyrus	8	-74	-20	7.12
	30	-68	-22	6.96
Lingual Gyrus	8	-78	0	6.75

Statistical significance according to FDR adjustment: verbal dual-task > verbal single-task; z = 2.3; Brain regions according to Harvard-Oxford Cortical Structural Atlas.

**Table 3:** Significant activation patterns (local maxima) for the spatial dual-task condition.

Brain Regions	Spatial Dual-Task > Spatial Single-Task			
	x	y	z	Z-score
Inferior Frontal Gyrus	-38	18	24	6.34
	-38	46	14	6.17
Middle Frontal Gyrus	38	34	26	7.23
	26	40	14	6.88
	30	48	16	6.68
	30	44	22	6.65
	38	54	16	6.42
	38	44	20	6.4
	-32	30	20	6.95
	-36	38	14	6.5
	-36	32	30	6.46
Frontal Orbital Cortex	-34	30	-2	5.56
Cingulate Gyrus	8	22	30	6.59
	32	2	44	6.48
Paracingulate Gyrus	-6	10	50	7.24
	-36	38	24	6.11
Lingual Gyrus	8	-76	2	8.03
	10	-74	-2	7.92
	14	-76	-10	7.68
	2	-74	2	7.6
	-8	-80	0	7.58
Juxtapositional Lobule Cortex	-4	6	52	7.24
	-28	0	50	7.1
Precentral Gyrus	-44	-4	44	6.96
Precuneus Cortex	2	-66	44	7.52
	-4	-68	46	7.19
	0	-68	56	6.93
	8	-68	38	6.93
	6	-60	52	6.81
	6	-66	50	6.76
Occipital Fusiform Gyrus	28	-70	-16	7.22
Lateral Occipital Cortex	-24	-74	42	6.39
	-28	-66	48	6.26
	-30	-68	40	6.15
	-22	-62	46	6.06
	-22	-58	36	5.91
	-28	-56	44	5.87
Left Thalamus	-14	-8	10	5.99
	-18	-2	22	5.73
	-14	2	6	5.71
	-14	-10	16	5.7
	-10	-20	10	5.63
Left Caudate	-16	-4	18	5.63
Frontal Operculum Cortex	-36	16	4	6.49
	-42	12	4	6.31
Insular Cortex	-30	24	2	5.54
Left Putamen	-26	12	4	5.1
	36	18	4	5.71
	30	24	2	5.51

Statistical significance according to FDR adjustment: spatial dual-task > spatial single-task; z = 2.3; Brain regions according to Harvard-Oxford Cortical Structural Atlas.

### Group comparisons

Compared to men, women showed regional increased network activation in the right anterior paracingulate gyrus as well as in the right prefrontal cortex and the left orbitofrontal cortex in the verbal dual-task (masked with the mean dual-task activation of women). In contrast, men did not show increased activation in any area when compared to women in the verbal dual-task (see Fig 3B and Table 4).

**Table 4:** Significant activation differences (local maxima) for the verbal dual-task condition (women > men).

Brain Regions	*Verbal Dual-Task > Verbal Single-Task; Women > Men*			
	x	y	z	Z-score
Paracingulate Gyrus	14	52	6	2.16
	16	50	0	1.76
Frontal Pole	30	46	0	1.76
	34	36	-8	1.36
Frontal Orbital Cortex	-30	32	-12	1.98
	-14	50	4	1.76

Statistical significance according to FDR adjustment: verbal dual-task > verbal single-task; z = 2.3; Brain regions according to Harvard-Oxford Cortical Structural Atlas.

Considering the spatial dual-task, men compared to women showed increased activation within the left intracalcarine cortex and left lateral occipital cortex in the spatial single-task and masked with the mean activation of men for the spatial dual-task. In contrast, women did not show increased activation in any region compared to men for the spatial dual-task related to the spatial single-task and masked with the mean activation for women in the spatial dual-task condition (Fig 3B, Table 5).

**Table 5:** Significant activation differences (local maxima) for the spatial dual-task condition (men > women).

Brain Regions	*Spatial Dual-Task > Spatial Single Task; Men > Women*			
	x	y	z	Z-score
Intracalcarine Cortex	-12	-98	26	4.07
Occipital Pole	-4	-90	26	2.96
Lateral Occipital Cortex	-10	-82	14	2.67
	-2	-98	30	4.07
	0	-100	24	2.01
	0	-102	18	1.69

Statistical significance according to FDR adjustment: spatial dual-task > spatial single-task; z = 2.3; Brain regions according to Harvard-Oxford Cortical Structural Atlas.

### Group differences in single-task conditions (compared to baseline)

Contrasts between single-tasks and baseline activation of the verbal single-task condition showed increased activation for men in the right postcentral gyrus, the right superior parietal lobe, the right planum temporale, the right precuneus, the left prefrontal cortex, the left superior occipital lobe, the left superior temporal lobe, the left orbitofrontal cortex, the left superior parietal lobe, the left amygdala, and the left hippocampus. Women did not show increased activation in any region for the verbal single-task. Women showed increased activation for the spatial single-task in the left inferior frontal lobe (pars triangularis). Men did not show any region of increased activation. For the working memory task, men showed increased activation in the precuneus and intracalcarine cortex compared to women. Women did not show increased activation for the working memory single-task (see Table 6 for the local maxima).

**Table 6:** Significant activation differences (local maxima) for the single-task conditions brain regions.

	x	y	z	Z-score
***Verbal Single-Task; Men > Women***				
Paracingulate Gyrus	28	-38	54	4.16
Frontal Pole	-18	62	12	3.66
Planum Temporale	50	-32	12	4.18
Lateral Occipital Cortex	-54	-68	24	4.01
Superior Temporal Gyrus	-38	-36	4	4.17
Left Amygdala	-20	-10	-16	4.17
Left Hippocamcus	-20	-10	-20	3.96
***Spatial Single-Task; Women > Men***				
Inferior Frontal Gyrus	-38	26	6	3.88
Frontal Pole	-6	64	-2	3.46
***Working Memory Single-Task; Women > Men***				
Precuneous Cortex	-2	-68	20	3.84
Intercalcarine Cortex	-4	-76	16	3.44

Statistical significance according to FDR adjustment; z = 2.3; Brain regions according to Harvard-Oxford Cortical Structural Atlas.

## Discussion

The aim of this study was to investigate differences in behavioral performance and functional cerebral activation between men and women during dual-tasking. For that, a sex-specific fMRI paradigm was developed. After appropriately developing the tasks and selecting participants in a fashion to eliminate possible difference in behavioral performance, differences in fMRI activation between verbal and spatial dual-tasks were observed between men and women. Based on fMRI data, we here thus provide first evidence for sex-related neurophysiological differences during dual-tasking, which are fully independent of the behavioral performance level.

Offer and Schneider [30] demonstrated that women are forced to complete two tasks at the same time ten hours more often a week than men what suggest a better ability to perform multitasking. General knowledge regarding sex differences is assumed because of differences in daily living between men and women concerning housework and child care [30]. Compared to this, behavioral multitasking studies already suggest no differences in multitasking [1].

One way to operationalize multitasking and possible sex differences is a dual-task paradigm [3]. As a methodological strength, in the current study age, education, attention, and executive function were matched between sexes, strongly suggesting that the observed sex-related activation differences with the tasks exist merely due to processing differences in the brain. Consistent with existing literature, we failed to identify differences in behavioral performance between women and men for dual-tasking [1]. Men and women also showed equal behavioral performance on the single-task level, in line with the study by Neubauer et al. [24] who used a similar paradigm.

We could not replicate the behavioral sex-related differences in working-memory tasks described by previous empirical studies, what is inconsistent with existing literature [18]. However, as others, we noted increased reaction times and decreasing accuracy in the dual-task conditions compared to the single-task conditions [4,9]. Comparable to the bottleneck theory we found increased reaction times for the secondary tasks. However, increased reaction times were also found for the primary task and not just for the secondary task, as predicted by Pashler [31]. Stelzel et al. [9] and Herath et al. [4], who reported similar results as the present study, interpreted this effect by motor interference as determinants of increased reaction times and errors, for the primary as well as for the secondary task. Importantly, these effects were not sex-specific.

In fMRI, women compared to men showed increased activation for the spatial single task condition, while men demonstrated higher activation in the verbal single task condition. These activation differences for the verbal and spatial single-task conditions are comparable to previous studies [22,25,32]. However, for dual-task conditions, women compared to men demonstrated increased activation for the verbal dual-task compared to the verbal single-task. Such difference between single and dual-task conditions was not observed in men. Parts of the frontal gyrus and the paracingulate yielded increased activation for women over men while performing two tasks simultaneously, if the primary task required processing of verbal stimuli. This greater regional activation lead to the same behavioral performance, supporting the interpretation that women activate the inferior frontal gyrus much more for the processing of verbal stimuli if they appear in a dual-task condition [33]. This result appears consistent with previous observations that women show increased and more bilateral activation patterns for verbal tasks [22,25].

For the spatial dual-task, activation in the spatial dual-task compared to the spatial single-task was increased in men over women, but vice versa there were no differences. This activation in areas of the occipital cortex implicated in visual processing might be related to analyzing the spatial stimuli [34]. One interpretation might refer to a more scrutinized analysis of spatial stimuli by men compared to women, if spatial processing is needed for dual-tasking, consistent with the findings of Semrud-Clikeman et al. [32], who have shown that visual attention plays a greater part for men in mental rotation tasks. This study also showed increased activation in the left middle occipital cortex in men while performing mental rotation tasks. In contrast to the present findings, this activation difference was associated with a better performance of men in mental rotation tasks, similar to previous studies showing associations between better performance and decreased activation [35].

These results partly also could contribute to the entities of neuronal efficiency vs. expertise. Parallel to comparable behavioral performance between sexes, men showed increased activation during the verbal single task, while women showed increased activation in the spatial single task, which may lead to the assumption that men process spatial tasks more efficiently while women do so for verbal tasks to achieve a similar cognitive performance (neuronal efficiency). However, the opposite was found for the more complex task, which included the additional load for working memory. Here, women showed increased activation in the frontal cortex during the execution of the verbal dual-task, while men showed increased activation in occipital areas during the spatial dual-task, despite comparable behavioral performance. Whilst for the performances in the single task condition a ceiling effect can be determined (verbal single-task ≥ 9.25 of max. 10 correct responses, mean = 9.74±0.22), a similar effect does not hold true for the dual task condition (verbal dual-task ≥2.40 of max. 10 correct responses, mean = 7.72±1.94). Therefore, it is thinkable that both sexes evolve different mental strategies to solve the more complex task, which might be based on prior expertise.

Some limitations of our study also need to be considered. Clearly, verbal and spatial dual-tasking should not be regarded as the only approach to assess sex-related differences in multitasking. It is therefore important to emphasize that this study only explains the difference between men and women in processing verbal and spatial tasks in combination with a secondary task. Further investigations thus are necessary to more precisely elaborate on sex related differences in multitasking.

## Conclusion

We here implemented verbal and spatial tasks together with a working memory task in a dual-task paradigm to assess sex-related differences in dual-tasking. No behavioral differences in accuracy or reaction times were noted, but activation differences for the verbal dual-task and the spatial dual-task were found between the sexes. Men and women varied regionally in functional activation in several areas. The fMRI findings suggested that women need an increased involvement of frontal regions to analyze verbal stimuli in a verbal dual-task condition, while men resort to an increased activation of visual areas to analyze the spatial stimuli in a spatial dual-task condition.

## Supporting information

S1 Table Minimal raw data of the study for all participants. (XLSX)
